# Value of Estimated Glomerular Filtration Rate and Albuminuria in Predicting Cardiovascular Risk in Patients with Type 2 Diabetes without Cardiovascular Disease

**DOI:** 10.1155/2018/8178043

**Published:** 2018-12-26

**Authors:** Thilak Weerarathna, Gayani Liyanage, Meththananda Herath, Miyuru Weerarathna, Isuru Amarasinghe

**Affiliations:** ^1^Professor in Medicine, Department of Medicine, Faculty of Medicine, University of Ruhuna, PO Box 70, Galle, Sri Lanka; ^2^Senior Lecturer in Pharmacology, Department of Pharmacology, Faculty of Medicine, University of Ruhuna, PO Box 70, Galle, Sri Lanka; ^3^Senior Lecturer in Medicine, Department of Medicine, Faculty of Medicine, University of Ruhuna, PO Box 70, Galle, Sri Lanka; ^4^Medical Student, Faculty of Medicine, University of Colombo, Sri Lanka; ^5^Lecturer in Pharmacology, Department of Pharmacology, Faculty of Medicine, University of Ruhuna, PO Box 70, Galle, Sri Lanka

## Abstract

**Introduction:**

Onset of nephropathy in patients with type 2 diabetes (T2DM) increases the cardiovascular disease (CVD) risk. Association of the parameters of diabetic nephropathy such as albuminuria and estimated Glomerular filtration rate (eGFR) with predicted CVD risk has not been studied in Sri Lankan patients with T2DM.

**Methods:**

In a cross-sectional study of patients who underwent single visit screening at a diabetes center in Sri Lanka, we obtained demographic and biochemical data. Those with urine albumin excretion over 30 mg/g creatinine were considered as having albuminuria, and eGFR was calculated using modified diet in renal disease (MDRD) formula. Ten-year coronary heart disease risk (CHDR) in all patients was calculated using United Kingdom Prospective Diabetes Study risk engine, and those with CHDR > 10% were considered as having high risk. Spearman correlation was used to study the association between eGFR and CHDR, and logistic regression analysis was carried out to study the association of albuminuria and eGFR with high (>10%) CHDR.

**Results:**

Of the patients with diabetes studied (n=2434), 64% (1563) were males. Mean (SD) age and duration of diabetes were 52 (11) and 9 (3) years, respectively. Normoalbuminuria, microalbuminuria, and macroalbuminuria were observed in 16.4%, 14.8%, and 68.7% of patients, respectively. Three hundred ninety-four (16.2%) patients had eGFR < 60 ml/min. Moderate correlation was observed between eGFR and predicted CHDR [r = (-0.4), P<0.01] and between eGFR and fatal CHDR (FCHDR) [r = (-0.5), P<0.01]. Independent t-test showed that patients with eGFR < 60 ml/min were older and had longer diabetes duration and lesser BMI compared to those who had eGFR > 60 ml/min (P < 0.01). On logistic regression, nephropathy according to eGFR became a strong predictor for high CHDR (OR; 3.497, 95% CI 2.08 to 5.87), and nephropathy according to albuminuria and both albuminuria and eGFR was not significant predictor of CHDR.

**Conclusions:**

Predicted CHDR shows a moderate and significant association with eGFR in patients with T2DM without symptomatic CVD. eGFR is a stronger predictor than albuminuria in predicting high CHDR in patients with T2DM. Intensification of CVD prevention measures should be done more confidently among patients with T2DM and reduced eGFR than in those with albuminuria alone.

## 1. Introduction

Type 2 diabetes mellitus (T2DM) is spreading as a pandemic [1]. Developing countries in the South Asian region including Sri Lanka are facing an enormous healthcare burden in managing patients with different acute and chronic complications of T2DM [2, 3]. Cardiovascular disease (CVD) and chronic kidney disease (CKD) are two major chronic complications of T2DM [4]. While CVD accounts for around 75% of deaths among individuals with T2DM, end stage renal disease (ESRD), due to diabetic nephropathy (DN), is one of the common indications for renal replacement therapy worldwide [5, 6]. Moreover, these two complications are closely interrelated as the biochemical and neurohumoral changes that occur in CKD are known to increase the CVD risk [7]

Guidelines laid down by the professional organizations such as the American Diabetes Association (ADA) recommend early identification of patients with high CVD risk in order to implement necessary therapeutic interventions as primary prevention measures to reduce their morbidity and mortality [8]. In clinical practice, this is done by calculating the risk of developing an adverse clinical event such as acute myocardial infarction of stroke during the next ten years using a risk calculator.

Several risk calculating tools are available to assess the CVD risk among patients with T2DM at primary care level [9]. UKPDS (United Kingdom Prospective Diabetes Study) risk engine is a risk scoring tool introduced after the landmark UKPDS trial to calculate the coronary heart disease risk (CHDR) in patients with T2DM without symptoms of CVD [10]. It uses several parameters including the age, gender, glycosylated hemoglobin level (HbA1c), and blood pressure to calculate the probability of developing adverse or fatal cardiovascular event over the next decade in a patient with T2DM [11].

DN is diagnosed by demonstration of albumin over 30mg/grams of creatinine in urine (albuminuria) or reduction of estimated glomerular filtration rate (eGFR) less than 60 ml/min using a standard formula. Studies carried out in the developed countries have shown that there is a considerable variation in the association of albuminuria and eGFR with cardiovascular morbidity and mortality [12, 13].

Although the association between DN and high CVD risk is well established in studies conducted in the developed countries, the intensity of association of a calculated CVD risk score with either albuminuria or estimated glomerular filtration rate (eGFR) has not been studied in developing countries. Demonstration of such an association and its relative strength would be useful for primary care physicians for therapeutic decision making when managing patients with T2DM in a resource poor setting. For example, if there is a strong association of one of the parameters over the other (albuminuria over eGFR or vice versa), primary care clinicians in the resource poor setting can more confidently commence primary preventive strategies for reduction of future CVD risk for patients under their care even without calculating the CHDR score which requires several parameters such as glycosylated hemoglobin level and lipoprotein levels.

There is a rising incidence of T2DM and associated burden of CVD and CKD Sri Lanka [14]. With this background, we aimed to study the association of a diabetes specific CHDR scoring tool, namely, UKPDS risk engine and albuminuria and eGFR, two commonly used tests to diagnose DN in patients with T2DM without symptomatic CVD.

## 2. Methods

This cross-sectional study used a database which included demographic and biochemical data of patients with T2DM who underwent single visit screening at a diabetes center in Southern Sri Lanka. Adult patients with T2DM over the age of 18 years who presented for single visit screening over three-year period from 2013 to 2017 were included. Patients with an established coronary artery, cerebrovascular, or peripheral arterial disease (those with history of coronary artery or cerebrovascular disease and those with ankle brachial blood pressure index less than 0.9); those with history of nondiabetic renal diseases, for example, due to connective diseases and obstructive uropathy or nephritic/nephrotic syndrome; and those with evidence of urinary tract infection in the urine deposits (pus cells more than 5 per high power field and/or positive nitrites in the dipstick test) were excluded. All pregnant mothers and patients with type 1 diabetes and those with clinical diagnosis of T2DM for less than one year were also excluded.

Data on age (obtained from the national identity card) and the duration of diabetes to the nearest year (verified from the clinic records) were obtained. Patients' weight and height were measured and their body mass index (BMI) was calculated. Blood pressure was recorded after at least five minutes' rest using an electronic instrument (Omron Corporation, Tokyo, Japan), as the mean of two readings taken five minutes apart.

Overnight fasting venous blood samples were collected to measure lipid profile. HbA1c level was estimated using high performance liquid chromatography (HPLC) method. After measuring serum creatinine level with Jaffe method, glomerular filtration rate (GFR) was estimated using modified diet in the renal disease (MDRD) formula. Urine albumin was measured with immune turbidimetry, and microalbuminuria and macroalbuminuria are categorized as less than 30 and more than 30 mg/g of creatinine, respectively. All chemical analyses including HbA1c, serum creatinine, and LDL cholesterol were performed in the laboratory attached to the diabetes center, and the same method of biochemical analysis was used throughout the study period.

### 2.1. Ethical Approval

Ethical clearance for the present study was obtained from the Institutional Ethics Committee of the Faculty of Medicine, University of Ruhuna. Written informed consent was obtained from all study subjects in the local language.

### 2.2. Statistical Analysis

All statistical analyses were performed using the Statistical Package for the Social Sciences. Unpaired t-test was used to compare continuous variables and Chi-square test was performed to compare categorical variables in different groups. Spearman correlation was used to study the association between eGFR with coronary heart disease risk (CHDR) and fatal CHDR (FCHDR). Logistic regression analysis was performed to detect the determinants for higher CHDR. P < 0.05 was considered statistically significant.

## 3. Results

Of the patients with diabetes studied (n=2434), 64 %  (1563) were males. Mean (SD) age and duration of diabetes were 52 (11) and 9 (3) years, respectively. Normoalbuminuria, microalbuminuria, and macroalbuminuria were observed in 16.4%, 14.8%, and 68.7% of patients, respectively. Three hundred ninety-four (16.2%) patients had eGFR < 60 ml/min. Descriptive data of the participants studied are shown in Table 1.

When the association of eGFR with CHDR was studied using Spearman correlation, an inverse correlation was noted between eGFR values and CHDR score values. Moderate correlation was observed between eGFR and CHDR [r = (-0.4), P<0.01] and between eGFR and coronary heart disease risk (FCHDR) [r = (-0.5), P<0.01].

Differences between the patients who had eGFR <60 ml/min and above the level were summarized in Table 2.

Results of independent t-test showed that patients with reduced eGFR were older and had longer diabetes duration and lesser BMI compared to those who had normal eGFR (P < 0.01). Further CHDR and FCHDR were significantly higher in the patients with diabetes with reduced eGFR (Mann–Whitney U test, P <0.01).

Differences of percentage of patients with nephropathy according to albuminuria and eGFR between the patients who had CHDR less than 10% and above 10% (high) are summarized in Table 3.

Percentage of patients who had lower eGFR was significantly higher among the patients who had high CVDR (P < 0.001). Further it was the same when nephropathy was decided by both eGFR and albuminuria. But the difference between the groups was not significant when only albuminuria was considered to define nephropathy.

When logistic regression was applied, presence of nephropathy according to eGFR became a strong predictor for high CHDR (OR; 3.49, 95% CI; 2.08 to 5.87), whereas presence of nephropathy according to albuminuria (OR; 1.316 95% CI; 0.99 to 1.71) and both albuminuria and eGFR (OR; 1.07 95% CI; 0.605 to 1.92) was not significant predictor of CHDR.

## 4. Discussion

This cross-sectional study revealed two significant findings on the association of albuminuria and eGFR with CHDR calculated by UKPDS risk engine among patients with T2DM without symptomatic CVD. Firstly, it revealed that there is a moderate but significant association of eGFR calculated with MDRD formula with the calculated CHDR. More importantly, it revealed that an eGFR value of less than 60 ml/min is relatively a stronger indicator of predicting CVD risk than a positive albuminuria report in this study population.

In clinical practice, diabetic nephropathy is diagnosed either when GFR falls to less than 60 ml/min or the urine albumin excretion exceeds 30 mg/gram creatinine. Association of both these parameters with macro vascular disease has been reported in different settings and revealed conflicting findings on their relative value as a surrogate of different types of CVD [15]. Most of these studies are prospective in design and are conducted in developed countries, and many have used incident CVD events as opposed to calculated CVD risk such as CHDR as in our study.

A study from Japan including 1002 T2DM patients with no history of CVD in four categories of CKD according to eGFR (≥90, 60-89, 30-59 and 15-29 ml/min) over five years with end points of an incident stroke and CHD reported that reduced eGFR is independently associated with incident CHD but not stroke. [16].

The FIELD (Fenofibrate Intervention and Event Lowering in Diabetes) study which included a larger population of 9,795 low-risk Caucasian patients with diabetes reported that both reduced eGFR and albuminuria were independent predictors of incident CVD over a follow-up period of five years (lower eGFR vs eGFR ≥ 90 ml/min/1.73 m^2^ was a risk factor for total CVD events (OR [95% CI] 1.14 [1.01-1.29] for eGFR 60-89 ml/min/1.73 m^2^; 1.59 [1.28-1.98] for eGFR 30-59 ml/min/1.73 m^2^; P < 0.001; adjusted for other characteristics) and albuminuria increased increasing total CVD (HR 1.25 [1.01-1.54] and 1.19 [0.76-1.85], respectively; P = 0.001 for trend) when eGFR ≥ 90 ml/min/1.73 m^2^ [17]). Another cross-sectional study from Italy, which evaluated 15,773 patients with type 2 diabetes, has reported that CVD risk increased linearly with eGFR decline and albuminuria and became significant for values < 78 mL/min/1.73 m^2^ and ≥ 10.5 mg/24 h, respectively. Total CVD showed an independent association with albuminuria alone (OR 1.20 [95% CI 1.08-1.33]), reduced eGFR alone (OR 1.52 [95% CI 1.34-1.73]), and both (OR 1.90 [95% CI 1.66-2.19]). In this study too, coronary artery diseases were associated predominantly with reduced eGFR alone, whereas cerebrovascular and peripheral events showed a stronger correlation with the albuminuric CKD phenotypes [12].

A Chinese study, which is similar in design to our study, conducted with 1401 in patients with T2DM and using UKPDS risk engine, has reported that CHDR was significantly increased with CKD stage (20.1%, 24.8%, and 34.3% in T2DM patients with no CKD, CKD Stage 1-2, and Stage 3-5, respectively; P < 0.001 for all). The stroke risk was also increased with CKD stage (8.6%, 12.7%, and 25.4% in T2DM patients with no CKD, CKD Stage 1-2, and Stage 3-5, respectively; P < 0.001 for all). This Chinese study however has not compared the value of albuminuria in the risk prediction in the study sample [7].

Our study findings are clinically relevant in the background of above-mentioned literature. Moderate association of predicted CHDR with declining eGFR in our study further strengthens the long held notion that progressive rise in serum creatinine level increases not only the incident CVD events but the predicted CHDR in patients with T2DM. Therefore, clinicians should intensify measures to reduce cardiovascular morbidity and mortality of diabetic patients with declining eGFR even without calculating their CHDR.

We also found that diagnosis of diabetic nephropathy with eGFR of less than 60 ml/min is better surrogate of high predicted CHDR than a single report of albuminuria. This finding has economical and practical implications as well. In the resource poor setting, estimation of serum creatinine is convenient and cheaper investigation than the testing and confirming albuminuria in a patient with diabetes. The latter can be false positive in several situations such as during a febrile illness and urinary tract infection or in the presence of severe hyperglycemia [18]. Guidelines recommend confirmation of albuminuria after demonstrating two out of three tests for urine albumin positive or carrying out urine albumin to creatinine ratio (ACR) [15]. Therefore confirmation of albuminuria cannot be done with a single report of positive albumin in urine and requires relatively expensive and time consuming procedure which cannot be done at a busy outpatient clinic. Moreover, some patients with diabetes are known to progress to nephropathy without having increased urine albumin excretion [19]. This category is known to have nonalbuminuric CKD and is postulated to have different pathophysiological mechanisms underlying the progression of CKD [20]. All these factors make albuminuria a relatively less robust indicator of nephropathy in patients with diabetes compared to eGFR although the former is an earlier finding in DN than the later. Therefore as a surrogate of CVD risk too, it can be argued that eGFR is a less expensive and more useful investigation compared to albuminuria in patients with T2DM.

In this study, we found a statistically significant association of diabetic nephropathy diagnosed with eGFR less than 60 ml/min compared to albuminuria with predicted CVD. Our finding make eGFR not only a more convenient and less expensive but a reliable and proven indicator of predicted CVD over albuminuria in patients with T2DM with no symptoms of CVD.

Having shown clinically useful findings on the value of eGFR and albuminuria on the predicted CVD risk in more than 2000 Sri Lankan patients with T2DM for the first time, our study has few limitations. The cross-sectional design is a major limitation. Also due to practical and financial constraints in the outpatient setting, we used a single positive report of albuminuria rather than confirming it with two out of three tests being positive or calculating the ACR.

However we excluded patients with urinary tract infection or hematuria in diagnosing albuminuria with appropriate measures.

In conclusion, we report that eGFR has a statistically significant, moderate association with predicted CHDR, and diabetic nephropathy diagnosed with eGFR has significantly higher odds than albuminuria alone in predicting high CVD risk calculated with UKPDS risk engine in patients with T2DM. We hope the latter finding would compel clinicians in the resource poor setting to intensify CVD prevention strategies upon diagnosis of diabetic nephropathy with reduced eGFR in preference to a single urine report positive for albumin in patients with T2DM. With rising incidence of T2DM and associated burden of CVD and CKD in Sri Lanka, our findings highlight the need for a meta-analysis of the studies conducted on this subject and/or a prospective, multicenter study on these two and perhaps other parameters of DN and cardiovascular outcomes.

## Figures and Tables

**Table 1:** Descriptive data on the sample (n=2434).

Variable	Mean (SD) or median (IQR)
Age (years)	52 (11)†
Duration of diabetes (years)	9 (3)†
BMI (Kg/m^2^)	24.4 (3.9) †
Systolic blood pressure (mmHg)	125 (17) †
Diastolic blood pressure (mmHg)	78 (8) †
HbA1c (%)	7.4 (0.7) †
eGFR (ml/min)	83 (22) †
Total cholesterol (mg)	197.1 (43.9) †
Low density lipoprotein (mg)	124.9 (34.7) †
Triglyceride (mg)	115.4 (51.3) †
High Density Lipoprotein (mg)	49.9 (9.9) †
CHDR	0.079 (0.050 to 0.129)‡
FCHDR	0.037 (0.018 to 0.072) ‡

BMI: body mass index, CHD: coronary heart disease risk, eGFR: estimated glomerular filtration rate, FCHD: fatal coronary heart disease risk.

† indicates mean (SD); ‡ indicates median (IQR).

**Table 2:** Differences between patients with and without nephropathy based on estimated glomerular filtration rate.

**Variable**	**Patients with eGFR > 60**	**Patients with eGFR < 60 **	**P **
	** ml/min**	**ml/min**	**value**
Age (years)	50 (10) †	63 (8) †	<0.01
Duration of disease (years)	8 (2) †	12 (4) †	<0.01
BMI	24.8 (4.0) †	22.4 (3.1) †	<0.01
CHDR	0.074 (0.046 to 0.116) ‡	0.128 (0.077 to 0.196) ‡	<0.01
FCHDR	0.031 (0.016 to 0.062) ‡	0.074 (0.046 to 0.116) ‡	<0.01

BMI: body mass index, eGFR: estimated glomerular filtration rate, CHDR: coronary heart disease risk, FCHDR: fatal coronary heart disease risk.

† indicates mean (SD); ‡ indicates median (IQR).

**Table 3:** Differences between patients with and without higher CHDR.

Variable	Patients with CHDR < 10%	Patients with CHDR > 10%	P value
Patients with nephropathy by eGFR	141 (9.6%)	251 (28%)	<0.001
Patients with nephropathy by albuminuria	1212 (82.2%)	76.7 (85.4%)	0.05
Patients with nephropathy by eGFR and albuminuria	109 (7.4%)	208 (23.2%)	<0.001

eGFR: estimated glomerular filtration rate, CHDR: coronary heart disease risk.

## Data Availability

The data used to support the findings of this study are available from the corresponding author upon request.
